# STING agonist 2’3’-cGAMP as an effective adjuvant for HPV16 peptide vaccine enhances anti-tumor immunity in TC-1 mice models

**DOI:** 10.3389/fcimb.2026.1798489

**Published:** 2026-06-18

**Authors:** Yina Cun, Rui Yang, Jie Dai, Xinwen Zhang, Lili Zhou, Li Shi, Jing Li, Haocheng He, Shuyuan Liu, Yufeng Yao

**Affiliations:** 1 Department of Immunogenetics, Institute of Medical Biology, Chinese Academy of Medical Sciences & Peking Union Medical College, Kunming, Yunnan, China; 2 School of Life Sciences, Yunnan University, Kunming, Yunnan, China; 3 School of Medical Sciences, Shanxi Medical University, Jinzhong, Shanxi, China; 4 Institute of Cancer Biology, Shanxi Medical University, Jinzhong, Shanxi, China; 5 Shanxi Center of Technology Innovation for Biomolecular Imaging & Precision Oncology, Shanxi Medical University, Jinzhong, Shanxi, China; 6 Yunnan Key Laboratory of Vaccine Research & Development on Severe Infectious Disease, Institute of Medical Biology, Chinese Academy of Medical Sciences & Peking Union Medical College, Kunming, Yunnan, China; 7 Department of Epidemiology and Statistics, West China School of Public Health and West China Fourth Hospital, Sichuan University, Chengdu, China

**Keywords:** adjuvant, HPV 16, peptide vaccine, STING agonists, therapeutic vaccine

## Abstract

**Background:**

Adjuvants are critical for enhancing vaccine immunogenicity. The agonists in cyclic GMP-AMP synthase (cGAS)-stimulator of interferon genes (STING) signaling pathway have demonstrated robust immune activation in preclinical models. Peptide vaccines targeting T cell epitopes of high-risk human papillomavirus (HPV) E6 and E7 represent a promising immunization strategy. To improve immunogenicity, we utilized the STING agonist 2’3’-cGAMP as an adjuvant and evaluated its ability to enhance immune responses and antitumor efficacy.

**Methods:**

The immunogenicity and efficacy of a candidate vaccine, consisting of the HPV16 E7_43–77_ peptide adjuvanted with 2’3’-cGAMP, were evaluated in established TC-1 tumor transplantation models with different initial tumor sizes (2–3 mm and 5–6 mm in diameter). Tumor-bearing mice received three weekly peritumoral subcutaneous vaccine doses. The effects on tumor suppression, antigen-specific cytotoxic T lymphocyte (CTL) response induction, and related immune mechanisms were investigated both *in vitro* and *in vivo*.

**Results:**

Immunization with the E7_43–77_ peptide adjuvanted by 2’3’-cGAMP significantly suppressed tumor growth and elicited high levels of Interferon (IFN)-γ and Granzyme B in CD8^+^ cytotoxic T lymphocytes. The vaccine also enhanced the differentiation of natural killer (NK) cells, dendritic cells (DCs), and M1-type macrophages, reduced Myeloid-derived suppressor cells (MDSCs), and increased INF-β levels, as well as promote lymphocyte infiltration and remodeling in tumor immune microenvironment (TME). Mechanistically, 2’3’-cGAMP promoted DC maturation, enhanced T cell proliferation and activation, and strengthened antigen-specific CTL responses by activating the STING-TBK1-IRF3 and STING-NF-κB pathways in peptide-loaded DCs.

**Conclusion:**

The STING agonist 2’3’-cGAMP serves as an effective adjuvant that enhances the therapeutic efficacy of an HPV16 peptide vaccine. These findings indicate its potential as a candidate therapeutic for HPV16 persistent infection and associated malignancies.

## Graphical Abstract

**Figure:**
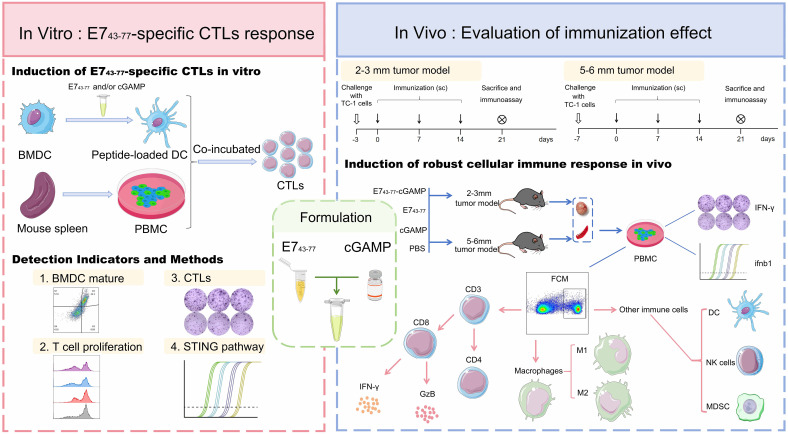

## Highlights

- STING agonist 2’3’-cGAMP enhances the efficacy of an HPV16 E7 peptide vaccine, leading to significant suppression of tumor growth in TC-1 mouse models.
- It induced a robust and effective immune response by promoting dendritic cell maturation, activating CD8^+^T cells, and increasing the production of IFN-γ and Granzyme B in CD8^+^ cytotoxic T lymphocytes, and facilitation lymphocyte infiltration and remodeling within the TME.
- The 2’3’-cGAMP adjuvanted HPV16 E7 peptide vaccine also enhanced the activation of NK cells and M1 macrophages through STING pathway activation.

## 1 Introduction

Cervical cancer one of the most common cancers among women globally. Persistent infection with high-risk human papillomavirus (hrHPV), especially type16 and type18 HPV, are the major factors leading to the occurrence and development of cervical cancer (Crosbie et al., 2013). Most HPV infections are asymptomatic and resolve spontaneously within 1 to 2 years. However, a minority of women may experience persistent HPV infection, which can potentially lead to cervical intraepithelial neoplasia (CIN) or cervical cancer (CC) (Blake and Middleman, 2017). Although the prophylactic HPV vaccines for cervical cancer has been rapidly applied at present, they cannot eliminate the existing HPV infections nor inhibit the development of cervical intraepithelial neoplasia lesions into malignant tumors (Van der Burg et al., 2001). The WHO preferred product characteristics for therapeutic HPV vaccines published in 2023, indicated that the development of therapeutic vaccines for HPV will serve as a crucial complement to the current approaches for preventing and treating HPV-related cancer (Organization, W.H, 2024). Therapeutic vaccines primarily targeting the oncoproteins E6 and E7 of hrHPV and aiming to induce HPV-specific CD8+ T cell-mediated cellular immune responses (Conageski, 2023). Therapeutic HPV vaccines are currently in early clinical development and the high immunogenicity and potential anti-tumor efficacy induced by the therapeutic vaccine is the key point to resolve.

The peptide vaccine targeting HLA-restricted hrHPV E6 and E7 epitopes is a promising vaccine development strategy (Ranasinghe and McMillan, 2025). T-cell epitope peptides can be taken up by antigen-presenting cells (APCs) and presented by MHC class I or II molecules without cleavage by proteasome. This induces highly reactive E6 and E7-specific cellular immune responses, generating virus-specific CD4^+^ and CD8^+^ cytotoxic T lymphocytes (CTLs) (Forman et al., 2012; Ranasinghe and McMillan, 2025). CTLs further secrete interferon-γ (IFN-γ), which control and eliminate infected or viral related tumor cells (Sasagawa et al., 2012). Although peptide vaccines have the advantages of high safety, strong stability and easy production, they are weakly immunogenicity, and optimized delivery system or combination with appropriate adjuvants are mostly necessary to enhance the immunogenicity and overall efficacy of the vaccine (Vonsky et al., 2019).

In recent years, many studies have demonstrated that the cyclic guanosine monophosphate-adenosine monophosphate synthase-stimulator of interferon genes (cGAS-STING) pathway activation is critical for anti-tumor immunity (Jiang et al., 2020; Barber, 2015; Kwon and Bakhoum, 2020). STING is an intracellular receptor that predominantly resided in the endoplasmic reticulum and is activated by binding of the agonist cGAMP (Ishikawa and Barber, 2008). STING signaling pathway induces the production of type I interferons (IFN-α and IFN-β) through a cascade of downstream signaling events such as activation TANK binding kinase 1 (TBK1), phosphorylation of interferon regulatory factor 3 (IRF3), and induction of nuclear factor kappa B (NF-κB) and serves as an essential component in initiating host innate immune response against infections, inflammation and tumor (Ishikawa and Barber, 2008; Sun et al., 2013; Wu et al., 2013; Wang et al., 2020). STING agonists are promising candidates for vaccine adjuvants and antitumor immune stimulants by activating this pathway. Some studies have attempted to use STING agonists as adjuvants for eliciting robust antitumor immunity of therapeutic vaccines and enhancing the specificity and effectiveness of some prophylactic vaccines (Li et al., 2013; Shen et al., 2025; Wang et al., 2016; Corrales et al., 2015). The 2’3’cyclic guanosine monophosphate-adenosine monophosphate (cyclic GMP-AMP, or cGAMP), a ligand for STING, is a potent natural agonist of STING. 2’3’-cGAMP has been applied as a vaccine adjuvant in some preclinic studies (Dorostkar et al., 2021; Van Dis et al., 2018; Wang et al., 2016).

In this study, we used the STING agonist 2’3’-cGAMP as an adjuvant in combination with the HPV16 E7_43–77_ peptide vaccine, aiming to explore whether 2’3’-cGAMP could induce a stronger immune response and enhance the anti-tumor effect of the peptide vaccine.

## 2 Materials and methods

### 2.1 Tumor cell lines and mice

TC-1 cell line, a murine C57BL/C(H-2b) lung epithelial cells co-transfected with Ras gene and E6 and E7 gene of HPV 16, were purchased from the Tumor Cell Bank of Chinese Academy of Medical Sciences. TC-1 cells were cultured in Roswell Park Memorial Institute (RPMI) 1640 medium (Gibco™ 21870076, USA) containing 10%FBS (Gibco™ 10099141C, USA) and 1% penicillin-streptomycin at 37°C in 5% CO_2_.

Female 6-8-week C57BL/6J mice were purchased from Institute of Medical Biology Chinese Academy of Medical Science. All mice were housed in a specific pathogen-free (SPF) environment.

### 2.2 Tumor model and immunization

1×10^5^ TC-1 cells in 50μL PBS mixed 50μL of Matrigel (Corning) were inoculated subcutaneously into the right back of each C57BL/6 mouse to establish TC-1 tumor model. Tumor measurements were taken three times weekly. Tumor volume was calculated using the formula: tumor volume (mm³) = 0.5 × length × width². Mice were euthanized humanely upon reaching a predefined ethical endpoint, defined as either a tumor length ≥20 mm or a tumor volume >1500 mm³.

On the 3^rd^ day following TC-1 implantation, the average diameter of the tumor was approximately 2-3mm. On the 7^th^ to 10^th^ day following TC-1 implantation, the average diameter of the tumor in mice was approximately 5-6mm. The tumor-bearing mice were randomly assigned to experimental groups to ensure intergroup homogeneity and received a 100 μL subcutaneous injection of the vaccine on the right flank once weekly for a total of three immunizations. Randomization was conducted using a computer-generated random number sequence to ensure balanced distribution of baseline tumor volumes across treatment groups. The antigen and adjuvant component consisted of 50 µg of HPV16 E7_43–77_ peptide and 5 µg of 2’3’-cGAMP (HY-100564, MedChemExpress Co.), respectively, per mouse. Mice were sacrificed seven days after the last immunization. At this time, spleens, blood and tumor were harvested to investigate the cell immunization response. All animal experiments have been approved by the Experimental Animal Ethics Committee of the Institute of Medical Biology, Chinese Academy of Medical Sciences (Approval No.: DWSP202311008).

HPV16 E7_43–77_ peptide were synthesized at Genscript ProBio (Nanjing, China).

### 2.3 BMDC maturation assay

Bone marrow (BM) cells were isolated from mice, counted, and seeded at a density of 1×10^6^ cells/mL in RPMI 1640 medium supplemented with 10% FBS, 20 ng/mL GM-CSF, and 10 ng/mL IL-4. Cells were cultured at 37 °C under 5% CO_2_, with cytokine replenishment every 48 hours. On day 5, non-adherent and loosely adherent cells were collected, centrifuged, and resuspended at 1×10^6^ cells/mL. The cells were then stimulated for 48 hours with E7_43-77_ plus cGAMP, E7_43-77_ alone, cGAMP alone, or PBS (control). The maturation of dendritic cells was assessed by flow cytometry using antibodies against CD11c, CD80, and CD86 and the cells were identified as peptide-loaded DCs.

### 2.4 T cell proliferation assay

Splenic lymphocytes were isolated from tumor bearing mice and resuspended at 1×10^7^ cells/mL in PBS containing 5% FBS. Cells were labeled with 5 μM CFSE (eBioscience, ThermoFisher) by gently mixed, and incubated at 37 °C in 5% CO_2_ for 10 min. The labeling reaction was quenched by cold RPMI 1640-medium supplemented with 10% FBS and washed. CFSE-labeled splenic lymphocytes were stimulated with 1 μg/mL anti-CD3 (145-2C11) and 1 μg/mL anti-CD28 (37.51) antibodies and subsequently cocultured with mature BMDCs that had been pre-stimulated under different conditions (PBS, peptide alone, cGAMP alone, or peptide + cGAMP) as described in Section 2.3, at effector-to target of 2:1 and 1:1 for 3 days. T cell proliferation was assessed by flow cytometry based on CFSE dilution.

### 2.5 Peptide-specific CTL response

For the *in vitro* assessment of CTL response, mature BMDCs loaded with synthetic peptide or other control stimulations, which prepared as described in section 2.3, were co-cultured with splenic lymphocytes isolated from naïve mice at a BMDC: lymphocyte ratio of 1:20 for 14 days. Recombinant murine IL-2 (20 ng/mL, Peprotech, USA) was supplemented on the 2^nd^ day. Half of the culture medium was replaced every 2-3 days. To enhance antigen-specific stimulation, an equal proportion of peptide-pulsed BMDCs were added on days 7^th^ day, 9^th^ and 12^th^ day of co-culture. On the 15^th^ day, antigen-specific T cells were harvested and designated as effector T cells. The effector T cells were subsequently used to investigate the IFN-γ-secreting.

The frequency of IFN-γ-secreting effector T cells was detected by the Mouse IFN-γ ELISPOT Plus Kit.

### 2.6 ELISPOT assay

Antigen-specific CTL response was evaluated by the IFN-γ secretion of T cell using enzyme-linked immunospot assay (ELISPOT). The ELISPOT assay was performed using the Mouse IFN-γ ELISPOT Plus Kit (Mabtech, Kista, Sweden) in accordance with the manufacturer’s instructions. In brief, cells were resuspended in serum-free ELISPOT medium and 2×10^5^ cells/well was added to the pre-coated ELISPOT plates, followed stimulus: HPV16 E7_43-77_ peptide (5 μg/mL), PHA (10 μg/mL, positive control), or medium alone (negative control). Plates were incubated for 24 h at 37 °C, 5% CO_2_. Post-incubation, cells were removed, and biotinylated detection antibody (R4-6A2-biotin, diluted in PBS with 0.5% FBS) and streptavidin-ALP (diluted in PBS with 0.5% FBS) were added successively to developed spots. The spots were enumerated using an ImmunoSpot^®^ Analyzer (Cellular Technology Limited, Shaker Heights, Cleveland, OH, USA).

### 2.7 IFN-I pathway analysis

The expression levels of genes associated with the cGAS-STING pathway and IFN-I pathway were assessed by quantitative real-time PCR (qRT-PCR) in BMDCs stimulated with either peptide or adjuvant. Total RNA was extracted from stimulated BMDCs (generated in Section 2.3) after 48 h of stimulation with PBS, E7_43-77_ peptide, 2’3’-cGAMP, or the combination using TRIzol reagent (Thermo Fisher Scientific, USA). cDNA synthesis was performed with 1 μg RNA using the TaKaRa PrimeScript™ RT cDNA Synthesis kit (TAKARA, China) and quantitative real-time PCR was performed using TB Green Premix (TAKARA, China) on the QuantStudio™ Real-Time PCR Detection System (Life Science, USA) according to the manufacturer’s instructions. Target gene expression was analyzed by normalizing the expression of house-keep gene *β-actin*. Primers used in this study were listed in supplementary (**Supplementary Table 1**).

### 2.8 Flow cytometry and antibodies

Single-cell suspensions were prepared from mouse spleens and tumors, which were treated with Zombie NIRTM Fixable Viability Kit and with FcR Blocking Reagent (both from BioLegend), followed by surface staining with specific antibodies. For intracellular staining, cells were fixed and permeabilized with the Foxp3/Transcription Factor Staining Buffer Set (eBioscience, ThermoFisher, USA) according to the manufacturer’s instructions. The following anti-mouse monoclonal antibodies were used: CD3-PerCP/Cyanine5.5, CD4-FITC, CD8-PE/Cyanine7, IFN-γ-PE, granzyme B (GzmB)-APC, CD107a-Brilliant Violet 510, perforin (PFN)-Brilliant Violet 421, CD25-APC, Foxp3-PE, CD11b-FITC, F4/80-Brilliant Violet 510, CD86-APC, CD206(MMR)-PE, Gr-1-PerCP/Cyanine5.5, CD11c-Brilliant Violet 605, MHC-II-PE/Cyanine7and NK1.1-Brilliant Violet 421. All antibodies were purchased from BioLegend (BioLegend Inc., San Diego, CA, USA).

### 2.9 Statistics

Statistical analyses were performed using GraphPad Prism software (version 9.0). Quantitative data are presented as the mean ± standard deviation (SD). Given that the data approximately follows a normal distribution, comparisons among multiple groups were conducted using one−way analysis of variance (ANOVA) followed by Tukey’s *post hoc* test. Significance levels were defined as follows: ns, *P* > 0.05; *, *P* < 0.05; **, *P* < 0.01; ***, *P* < 0.001; ****, *P* < 0.0001.

## 3 Results

### 3.1 E7-peptide adjuvanted with 2’3’-cGAMP elicits robust cellular immune responses *ex vivo*

**Figure 1:**
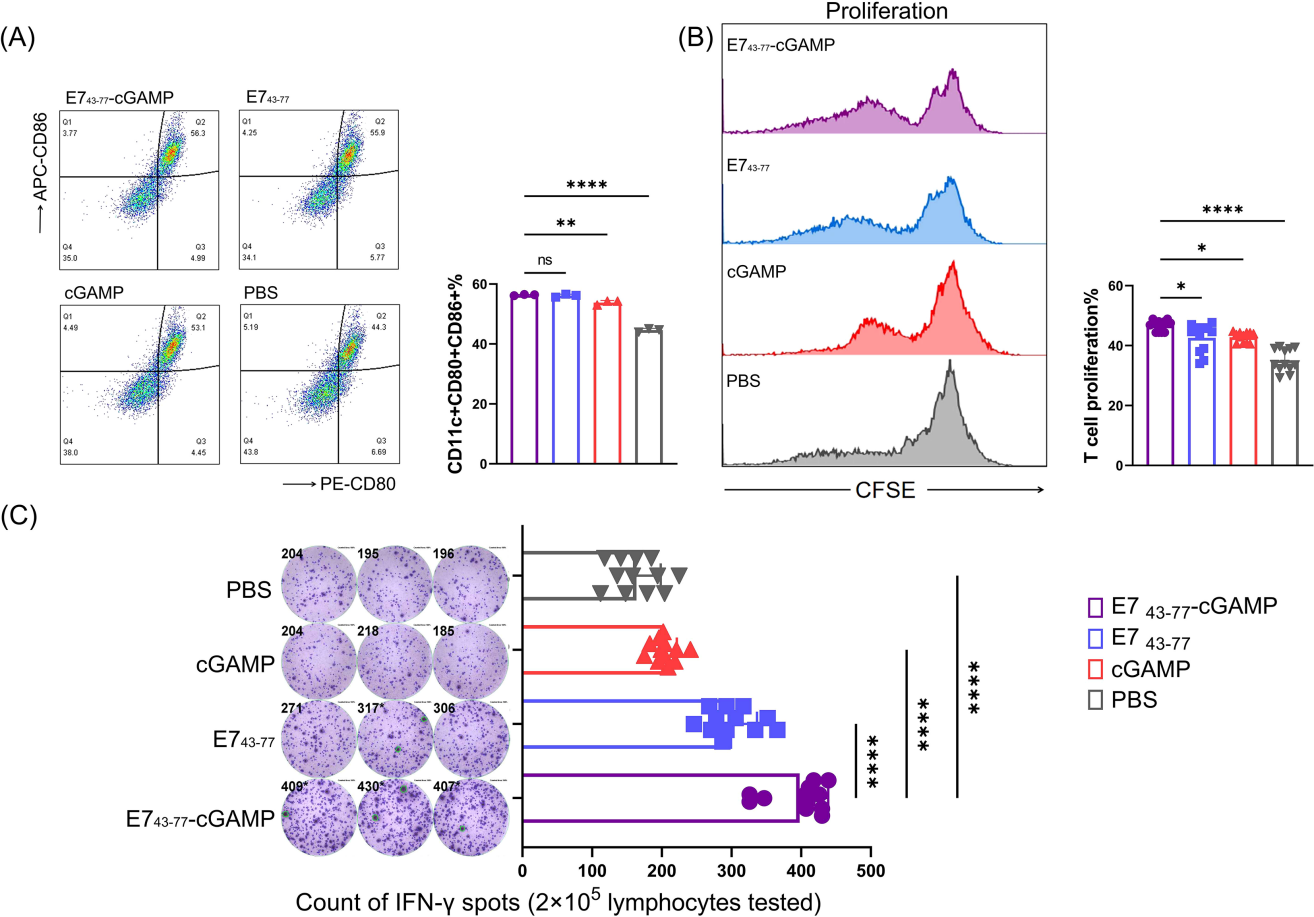
HPV16 E7_43–77_ peptide combined with 2′3′-cGAMP promotes the **(A)** maturation of BMDCs; **(B)** T cell proliferation; and **(C)** IFN-γ secreted by antigen-specific T lymphocytes induced *in vitro*. Note: Significance levels were defined as follows: ns, *P* > 0.05; *, *P* < 0.05; **, *P* < 0.01; ****, *P* < 0.0001.

The immunogenicity of the candidate vaccine comprising the HPV16 E7 _43–77_ peptide adjuvanted with 2’3’-cGAMP was initially evaluated *Ex vivo*. BMDCs stimulated with the peptide, the adjuvant, or their combination exhibited enhanced maturation, as indicated by significantly upregulated expression of CD80 and CD86 (**Figure 1A**). Notably, co-stimulation with E7 _43–77_ peptide and 2’3’-cGAMP elicited the most robust proliferation of antigen-specific T cells among all treatment groups (**Figure 1B**). Moreover, when BMDCs loaded with the E7 _43–77_ peptide were co-cultured with mouse splenic lymphocytes to generate the cytotoxic T lymphocytes (CTLs), CTLs generated in the combination group secreted significantly higher levels of IFN-γ compared to those from control groups (**Figure 1C**).

**Figure 2:**
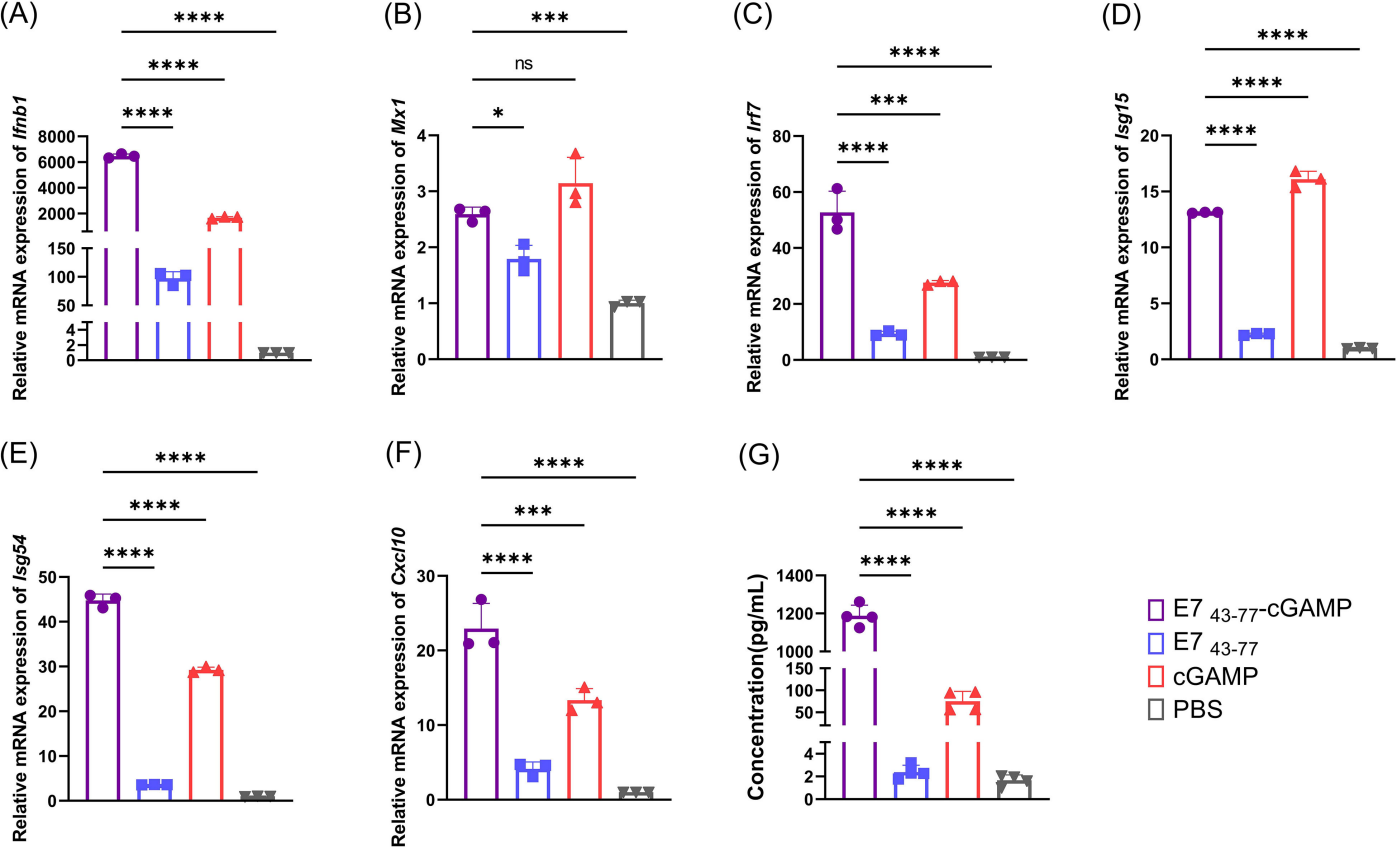
HPV16 E7_43–77_ peptide combined with 2′3′-cGAMP elicited the the expression of genes associated with cGAS-STING-pathway in peptide-loaded BMDCs: **(A)** ifnb1; **(B)** Mx1; **(C)** Irf7; **(D) **Isg15; **(E)** Isg54; **(F)** Cxcl10; as well as the secretion level of **(G)** IFN-β in the supernatant of BMDC cultures. Note: Significance levels were defined as follows: ns, *P* > 0.05; *, *P* < 0.05; ***, *P* < 0.001; ****, *P* < 0.0001.

To investigate the underlying mechanism, activation of the cGAS–STING pathway was assessed by quantifying the expression of downstream target genes in stimulated BMDCs. qPCR analysis revealed that treatment with 2’3’-cGAMP, either alone or in combination with the E7 _43–77_ peptide, significantly upregulated the mRNA expression levels of *Ifnb1*, *Isg15*, *Isg54*, *Mx1*, *Irf7* and *Cxcl10* (**Figure 2**). Notably, co-treatment with 2’3’-cGAMP and E7_43–77_ peptide further enhanced the expression of *Ifnb1*, *Irf7*, *Isag54* and *Cxcl10* genes relative to 2’3’-cGAMP treatment alone (**Figure 2**). Consistent with these transcriptional changes, IFN-β secretion into the culture supernatant was significantly increased in the combination treatment group (**Figure 2G**), confirming robust activation of the STING pathway.

### 3.2 E7-peptide adjuvanted with 2’3’-cGAMP effectively inhibits tumor growth

**Figure 3:**
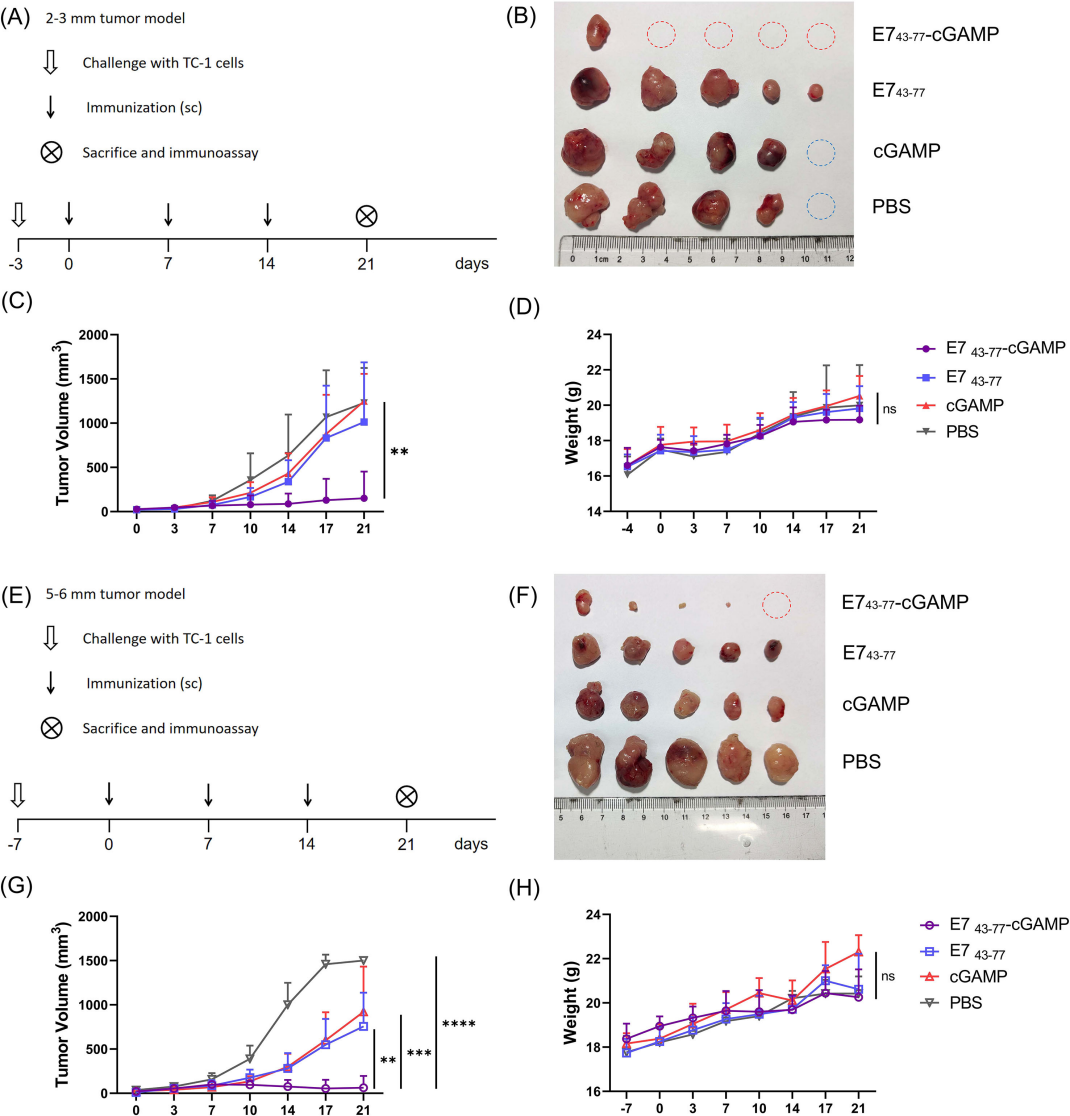
Effects of HPV16 E7_43–77_ adjuvanted with 2′3′-cGAMP on tumor growth in **(A–D)** the 2–3 mm TC-1 tumor mouse model and **(E–H)** the 5–6 mm TC-1 tumor mouse model; **(A, E)** depict the immunization schedule. Mice were euthanized when tumor volume exceeded the predefined ethical limit. Blue circles denote mice that were euthanized upon reaching the ethical endpoint, and thus no tumor was collected at the experimental endpoint; red circles indicate mice that were tumor-free at the experimental endpoint. Note: Significance levels were defined as follows: ns, *P* < 0.05; **, *P* < 0.01; ***, *P* < 0.001; ****, *P* < 0.0001.

The anti−tumor efficacy of the E7 _43–77_ peptide adjuvanted with 2’3’-cGAMP was evaluated in the TC-1 transplantation tumor models with initial diameters of 2–3 mm and 5–6 mm. The results showed that in the 2–3 mm tumor model, the tumor growth of E7_43-77_-cGAMP group was significantly inhibited (**Figures 3A–D**), with complete regression observed in four mice (**Figure 3B**). In the 5–6 mm tumor model, the E7_43-77_-cGAMP also exhibited a notable inhibitory effect on tumor progression (**Figures 3E–H**), leading to complete tumor clearance in one mouse (**Figure 3F**). Notably, no significant differences in body weight changes were observed between the experimental and control groups throughout the immunization period (**Figures 3D, H**), indicating that both 2’3’-cGAMP and the E7_43–77_ peptide are well tolerated and have no adverse effects on normal mouse growth.

### 3.3 E7-peptide adjuvanted with 2’3’-cGAMP potentiates anti-tumor immunity *in vivo*

**Figure 4:**
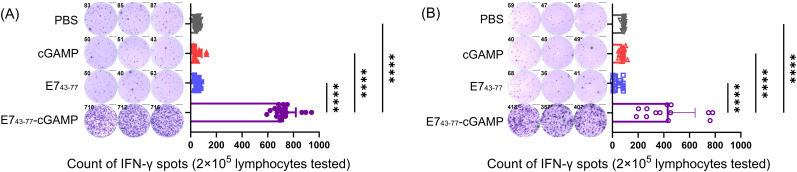
IFN-γ secreted by antigen-specific CTLs of spleen lymphocytes measured by ELISPOT **(A)** 2–3 mm TC-1 tumor mouse model; **(B)** 5–6 mm TC-1 tumor mouse model. Note: Significance levels were defined as ****, *P* < 0.0001.

Consistent with the *ex vivo* findings, vaccination with the E7_43–77_ peptide formulated with 2’3’-cGAMP elicited a robust antigen-specific cytotoxic T lymphocyte response *in vivo*, as quantified by IFN-γ ELISpot assay using splenocytes isolated from immunized mice. In both the 2–3 mm and 5–6 mm tumor models, the level of IFN-γ secretion by splenic lymphocytes in the E7_43-77_-cGAMP group was significantly higher than that in control groups (**Figures 4A, B**), correlating with enhanced inhibition of tumor growth.

**Figure 5:**
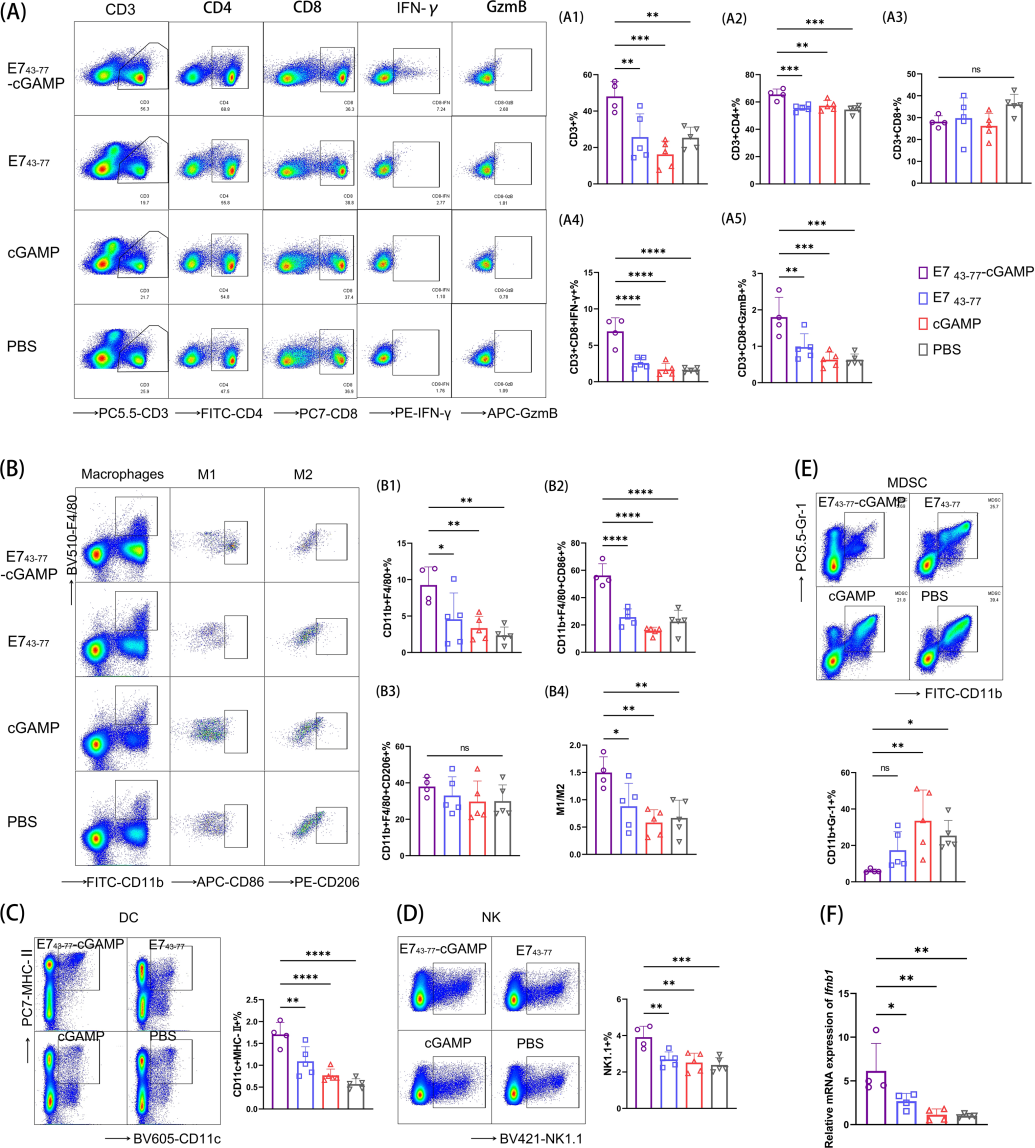
The proportion of various cells in spleen lymphocytes. Proportion of **(A)** CTLs, such as **(A1)** total T cells, **(A2)** CD4+T cells, **(A3)** CD8+T cells, **(A4)** CD8+IFNγ+ cells, and **(A5)** CD8+GzmB+ cells; **(B)** Macrophages, including **(B1)** total macrophage cells, **(B2)** M1-phenotype macrophage, **(B3)** M2-phenotype macrophage ,and **(B4)** the ratio of M1 to M2; **(C)** DCs; **(D)** NK cells; **(E)** MDSCs; **(F)** mRNA expression level of ifnb1 in spleen. Significance levels were defined as follows: ns, *P* > 0.05; *, *P* < 0.05; **, *P* < 0.01; ***, *P* < 0.001; ****, *P* < 0.0001.

Further immunophenotypic analysis of splenocytes from the 5–6 mm tumor model revealed that E7_43-77_-cGAMP vaccination substantially remodeled the immune composition (**Figure 5A**). In the E7_43-77_-cGAMP group, the proportion of CD3^+^ T cells significantly increased (**Figure 5A1**), accompanied by significant expansions of IFN-γ^+^ and Granzyme B^+^ (GzmB^+^) cells within the CD8^+^ T cell compartment (**Figures 5A4, A5**), indicative of potent CTL activation and functional differentiation. Moreover, the proportions of macrophages (**Figure 5B**), DCs (**Figure 5C**), and natural killer (NK) cells (**Figure 5D**) were all significantly elevated in the E7_43-77_-cGAMP group, with macrophages exhibiting a pronounced shift toward the pro-inflammatory M1 phenotype (**Figure 5B**). Concurrently, the proportion of MDSCs was markedly reduced (**Figure 5E**). Collectively, these data indicated that the application of 2’3’-cGAMP as an adjuvant in E7_43–77_ peptide vaccine and not only induces a strong CTL response, but also comprehensively enhances the anti-tumor immune capacity of the host by raising the activation or differentiation of NK cells, DC and M1-type macrophages, while suppressing MDSCs accumulation.

In addition, the mRNA expression level of *Ifnb1* in splenic lymphocytes of mice was significantly increased in the E7_43-77_-cGAMP group (**Figure 5F**), further substantiating the critical involvement of the STING–type I interferon (IFN-I) signaling axis in mediating the observed antitumor immunity.

### 3.4 Vaccination with a 2’3’-cGAMP-adjuvanted peptide vaccine reprograms the tumor immune microenvironment

**Figure 6:**
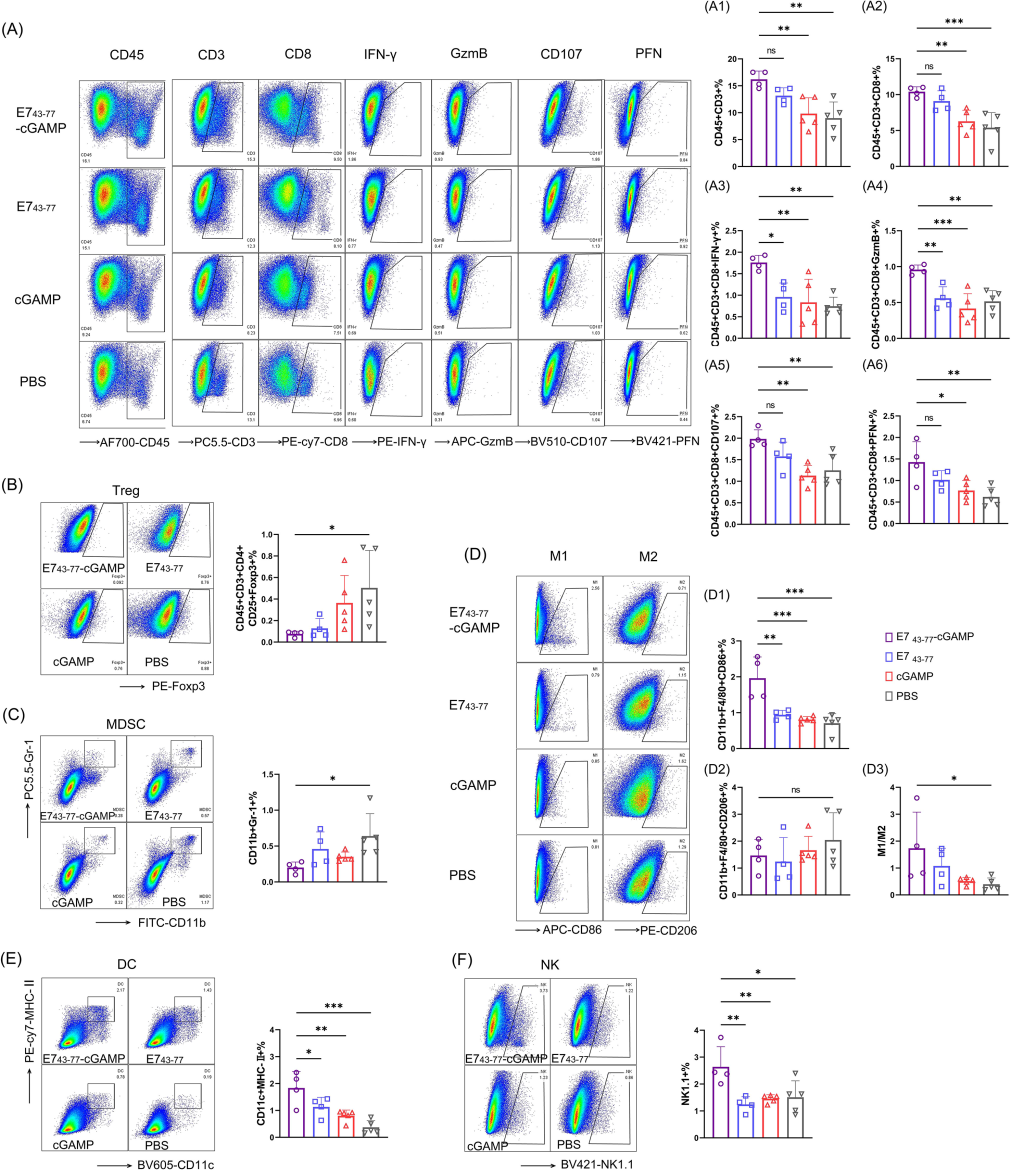
The proportion of lymphocytes infiltrated in tumors. Proportion of TILs **(A)** CTLs; such as **(A1)** total infiltrated T cells; **(A2)** CD8+ T cells; **(A3)** CD8+IFNγ+cells; degranulation markers of cytotoxic granules **(A4)** CD8+GzmB+, **(A5)** CD8+CD107+ and **(A6)** CD8+PFN+cells; **(B)** Tregs; **(C)** MDSCs; **(D)** Macrophages, compromised **(D1)** M1-phenotype macrophage, **(D2)** M2-phenotype macrophage and **(D3)** the ratio of M1 to M2; **(E)** DCs; and **(F)** NK cells. Significance levels were defined as follows: ns, *P* > 0.05; *, *P* < 0.05; **, *P* < 0.01; ***, *P* < 0.001.

To evaluate the impact of 2’3’-cGAMP-Adjuvanted Peptide Vaccine on the tumor immune microenvironment (TME), we comprehensively characterized the composition and functional status of immune cell populations infiltrating the TME in the 5–6 mm tumor model. Compared with control groups, the combination vaccine significantly increased the infiltration of tumor-infiltrating T lymphocytes, particularly CD8^+^ T cells, and markedly enhanced the production of key effector molecules, including IFN-γ, GzmB, CD107a, and perforin (PFN), collectively indicating augmented cytotoxic T-cell activity (**Figure 6A**). Concurrently, the vaccine significantly promoted the recruitment of NK cells and DCs into the tumor (**Figures 6E, F**). Furthermore, tumor-associated macrophages (TAMs) exhibited a phenotypic shift toward an M1-like pro-inflammatory polarization state (**Figure 6D**). In contrast, immunosuppressive cell populations, including MDSCs and regulatory T cells (Tregs), were substantially reduced following combination treatment (**Figures 6B, C**).

## 4 Discussion

The increasing global burden of HPV-associated malignancies underscores the urgent need for effective therapeutic vaccine strategies. Recent advances in cancer immunotherapy or therapeutic vaccine have highlighted the pivotal role of cell-mediated immune responses in clearing persistent HPV infection and preventing neoplastic progression (Ranasinghe and McMillan, 2025). Although several therapeutic HPV vaccines have entered clinical trials (Messa and Loregian, 2022), enhancing their immunogenicity remains a critical challenge.

In the present study, we evaluated the therapeutic efficacy of an HPV16 E7 _43–77_ peptide-based vaccine formulated with the STING agonist 2’3’-cGAMP using TC-1 tumor-bearing mouse models representing early-stage (2–3 mm) and established (5–6 mm) lesions. The HPV16 E7 _43–77_ peptide, which encompasses one CD8 T cell epitope (E7 _49-57_), two CD4 T cell epitopes (E7 _43–77_ and E7 _50-62_) and three distinct T-helper (Th) epitopes (E7 _50-62_, E7 _43-77_, E7 _35-50_) (Yang et al., 2019; Van der Burg et al., 2001), was selected for its demonstrated capacity to elicit cellular responses. Although peptide vaccines feature homogeneous antigenic components with highly purified, stable and easy to produce, but exhibit relatively weak immunogenicity, necessitating the incorporation of potent adjuvants to elicit effective adaptive immunity (Pulendran et al., 2021). Studies have demonstrated that peptides formulated with adjuvant such as CpG oligodeoxynucleotide (CpG), polyinosinic-polycytidylic acid (Poly I:C), 1,2-dioleoyl-3-trimethylammonium-propane (DOTAP) elicited robust antitumor cellular immunity (Kinkead et al., 2018; Zhu et al., 2008).

The cGAS-STING pathway has emerged as a key mediator of innate and adaptive immunity primarily through the induction of type I interferon (IFN) responses. This pathway functions as a cytosolic DNA sensor that is activated upon disruption of cellular homeostasis. cGAS recognizes accumulated cytoplasmic DNA and synthesizes the second messenger cyclic GMP-AMP (cGAMP), which binds to and activates the stimulator of interferon genes (STING). STING activation subsequently leading to the phosphorylation of the transcription factors 3 (IRF3) and nuclear factor kappa B (NF-κB), culminating in the production and secretion of type I interferons (IFNs) (Sun et al., 2013). Beyond its well-established role in innate immune initiation, the STING pathway serves as a critical bridge to adaptive immunity by enhancing T cell priming, effector function, and immunological memory, while also synergizing with antigen presentation. STING activation directly potentiates T cell effector functions. Wang et al (Wang et al., 2024). demonstrated that the STING agonist diABZl activates both the STING pathway and TCR signaling cascades, thereby enhancing TCR-T cells cytotoxicity and promoting tumor cell apoptosis. Similarly, Ugur et al (Uslu et al., 2024). reported that STING activation improves the function of chimeric antigen receptor T cell (CART), induces memory T cell formation and promote immune cell infiltration into tumors, ultimately augmenting the CART efficacy against solid tumors. In addition to its direct effects on T cells, STING activation promotes DC maturation, characterized by upregulated expression of MHC molecules, co-stimulatory ligands (such as CD80/CD86), and chemokines (such as CXCL9/10), thereby facilitating antigen cross-presentation and cytotoxic T lymphocyte induction (Dane et al., 2022). Furthermore, STING signaling contributes to remodeling the immunosuppression TME by increasing immune cell infiltration and suppressing Tregs and MDSCs (Wang et al., 2024, 2025; Ribeiro et al., 2024). Additionally, STING activation also induces the secretion of some pro-inflammatory cytokines, such as IL-18, which plays an important role enhancing cytotoxicity and upregulating FAS ligand expression, thereby eliciting anti-tumor immunity and sustaining T cell function (Uslu et al., 2024). Given these multifaceted roles in orchestrating anti-tumor immunity, the cGAS-STING pathway has aroused significant interests as a therapeutic target (Dane et al., 2022). Numerous cGAS-STING agonists have demonstrated promising efficacy in preclinical models, both as standalone therapies and as vaccine adjuvants capable of eliciting robust protective immunity against infectious diseases (Shen et al., 2025) and cancer (Decout et al., 2021). Several agonists, including ADU-S100 (Chelvanambi et al., 2021), SYNB1891 (Luke et al., 2023), MK-1454 (Chang et al., 2022), E7766 (Kim et al., 2021), and BMS-986301 (Feng et al., 2020), have advanced to clinical trials for cancer therapy, underscoring the translational potential of targeting this pathway.

As a natural STING agonist, 2’3’-cGAMP has been approved for clinical investigation. In the present study, we utilized 2’3’-cGAMP as an adjuvant in combination with the therapeutic peptide vaccine E7 _43-77_. Our findings demonstrate that co-administration of E7 _43–77_ adjuvanted with 2’3’-cGAMP elicited a robust and functionally effective immune response, resulting in significant tumor growth inhibition and complete tumor regression in 20–80% of treated mice. This therapeutic effect was accompanied by marked increases in IFN-γ^+^ and Gzm B^+^ CD8^+^ T cells, indicating enhanced CTL functionality. Notably, adjuvantation with 2’3’-cGAMP not only enhanced antigen-specific CTL responses but also broadly remodeled the immune composition in both spleen and TME. We observed increased infiltration and enhanced cytotoxic functionality of CD8+T cell within the TME, which upregulated expressed CD107a, IFN-γ, Granzyme B, and perforin, as well as increased differentiation of NK cells, DCs, and TAMs toward M1-polarized pro-inflammatory phenotype. Whereas the frequencies of immunosuppressive cell populations, including MDSCs and Tregs, were significantly reduced. These cellular changes were paralleled by elevated IFN-β expression, consistent with activation of the STING–IFN-I axis. *In vitro* mechanistic studies further revealed that 2’3’-cGAMP induced DC maturation via both STING–TBK1–IRF3 and STING–NF-κB signaling pathways, leading to enhanced T cell proliferation and activation. Collectively, these results indicated that E7 _43–77_ peptide combined with 2’3’-cGAMP activates both the innate and the adaptive immune response. This activation promotes multifaceted reprogramming of the tumor microenvironment, characterized by coordinated immunological changes that reflect a functional shift from an immunosuppressive to an immunostimulatory milieu, thereby significantly enhancing the antitumor efficacy.

The capacity of STING agonists to augment vaccine efficacy has been increasingly recognized. In 2015, cyclic dinucleotides (CDNs) were first employed as STING activation adjuvants in tumor cell vaccines (Fu et al., 2015). Subsequent studies have validated the effectiveness of STING agonists as vaccine adjuvants in various tumor models. Liu et al. developed a novel STING agonist, CF501, and demonstrated that the CF501 adjuvanted RBD-Fc vaccine induced robust and durable neutralizing antibody and T cell responses in mice, rabbits and nonhuman primates (Liu et al., 2022). Similarly, administration of STING agonists ADU-S100 in melanoma models increased the infiltration of CD8^+^T cells and CD11c^+^ DCs into TME, thereby enhancing the antitumor cytotoxicity (Chelvanambi et al., 2021). A small molecule STING agonist diABZI has also been reported to induce durable regression of established colon tumors (Ramanujulu et al., 2018). Our study extends these observations by demonstrating that 2’3’-cGAMP can serve as an effective adjuvant for a therapeutic HPV peptide vaccine, eliciting both local and systemic immune reprogramming.

The potent anti-tumor immunity observed herein is likely attributable to the integrated effects of STING activation on multiple immune compartments. As key regulators of T cell priming, DCs can also be activated by tumor cell-derived STING agonists following phagocytosis of tumor cells and subsequent tumor-associated antigen transport. STING-stimulated DCs acquire the capacity to prime and durable antigen-specific CTL and Th1 antitumor immune responses (Ribeiro et al., 2024). Simultaneously, IFN-I act directly on CD8^+^ T cells to promote their activation, proliferation, and cytotoxic activity within the tumor microenvironment, which enhanced the cancer immunosurveillance of host (Lu et al., 2019). Furthermore, STING-mediated suppression of MDSCs and Tregs, coupled with enhanced NK cell and M1 macrophage infiltration, contributes to a more permissive immunological milieu for tumor elimination. These synergistic actions underscore the potential of STING-targeted adjuvants to overcome immune evasion mechanisms that limit the efficacy of peptide-based cancer vaccines. Consequently, therapeutic strategies aimed at augmenting IFN-I signaling in T cells, such as those utilizing STING agonists, represent a promising avenue for improving cancer immunotherapy. Further investigation is warranted to optimize delivery systems that minimize systemic toxicity while maximizing antigen-specific immunity, potentially advancing the development of next-generation combination immunotherapies.

Despite these promising findings, we acknowledge several limitations. The current study lacks protein-level validation of key STING pathway components (e.g., phosphorylated TBK1 and IRF3) and functional confirmation using STING inhibitors or genetic knockout models to definitively establish pathway dependency. Additionally, histological analysis of treated tumors to assess immune infiltration and architectural changes within the TME was not performed. Future investigations incorporating these approaches will be essential to corroborate our transcriptional findings and to delineate the precise mechanisms by which 2’3’-cGAMP enhances anti-tumor immunity. Moreover, optimizing vaccine delivery systems to minimize systemic toxicity while maximizing antigen-specific responses remains a critical priority for translational advancement.

## 5 Conclusions

In conclusion, our study demonstrates that the natural STING agonist 2’3’-cGAMP functions as a potent adjuvant for an HPV 16 E7 peptide vaccine, eliciting robust eliciting robust and coordinated innate and adaptive immune responses that effectively suppress tumor growth. These findings support further exploration of STING-targeted combination strategies for the treatment of HPV-associated malignancies and other cancers amenable to immunotherapy.

## Data Availability

The original contributions presented in the study are included in the article/**Supplementary Material**. Further inquiries can be directed to the corresponding authors.
