# Supplementary material for: STING agonist 2’3’-cGAMP as an effective adjuvant for HPV16 peptide vaccine enhances anti-tumor immunity in TC-1 mice models
Source: Front Cell Infect Microbiol. 2026 Jun 18;16:1798489. doi: 10.3389/fcimb.2026.1798489 (PMC13323254; doi:10.3389/fcimb.2026.1798489)
Supplement: Supplementary file 1 [file DataSheet1.pdf]

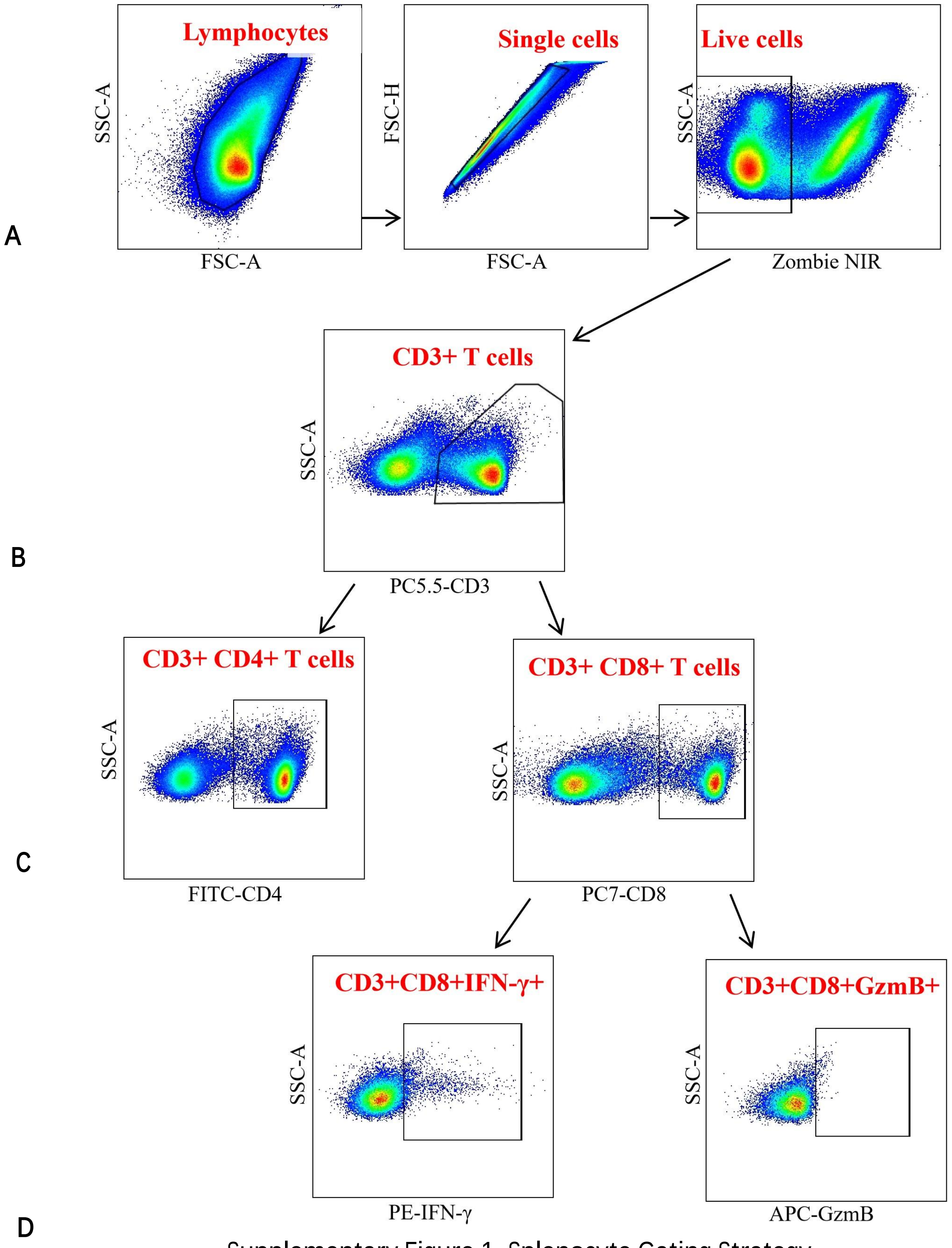

Supplementary Figure 1. Splenocyte Gating Strategy

(A) Forward scatter area (FSC-A) vs. height (FSC-H) plot, doublets and cell aggregates were excluded to select single cells (based on FSC-A/FSC-H correlation). Live cells were identified by excluding dead cells (stained with Zombie NIRviability dye) via a viability dye (Zombie NIR) vs. FSC-A plot. (B) CD3 T cells were selected using anti-CD3 (PC5.5) staining (CD3 vs. SSC-A plot). (C) CD3CD4 T cells: From the CD3 gate, CD4 T cells were identified via FITC-conjugated anti-CD4 staining (CD4 vs. SSC-A plot).(D)IFN-γ production: Within the CD3CD8 gate, IFN-γ-producing cells were detected via PE-conjugated anti-IFN-γ staining (IFN-γ vs. SSC-A plot).Granzyme B (Gzmb) expression: Within the CD3CD8 gate, Gzmb-expressing cells were detected via APC-conjugated anti-Gzmb staining (Gzmb vs. SSC-A plot).

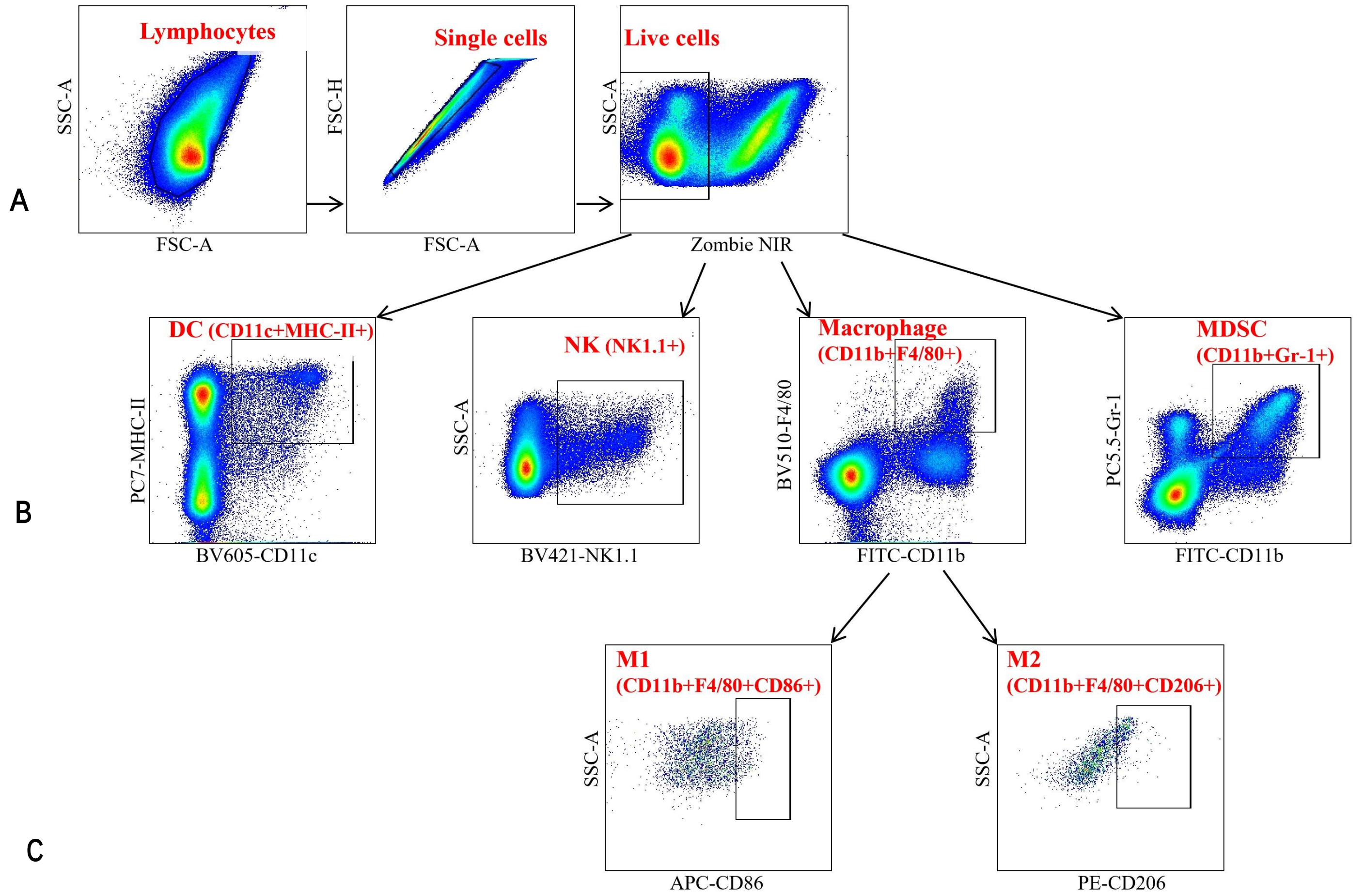

Supplementary Figure 2. Myeloid Cell Gating Strategy

(A) Initial debris and doublet exclusion using FSC-A/FSC-H and SSC-A/SSC-W parameters. Live/dead discrimination using a viability dye. Live single cells were further analyzed to identify specific myeloid lineages based on characteristic scatter and surface marker expression:(B) DC: Identified as CD11c+ and MHC-II+ within the live gate (BV605-CD11c vs. PC7-MHC-II), NK cells: Identified as NK1.1 (BV421-NK1.1 vs. SSC-A), Macrophages: Identified as CD11bF4/80 (FITC-CD11b vs. BV510-F4/80), and MDSC: Identified as CD11bGr-1 (FITC-CD11b vs. PC5.5-Gr-1). (C) Macrophage Polarization (M1/M2): Further phenotyping was performed on the CD11bF4/80 macrophage gate to distinguish between: M1 Macrophages: Identified as CD86 (APC-CD86 vs. SSC-A) and M2 Macrophages: Identified as CD206 (PE-CD206 vs. SSC-A).

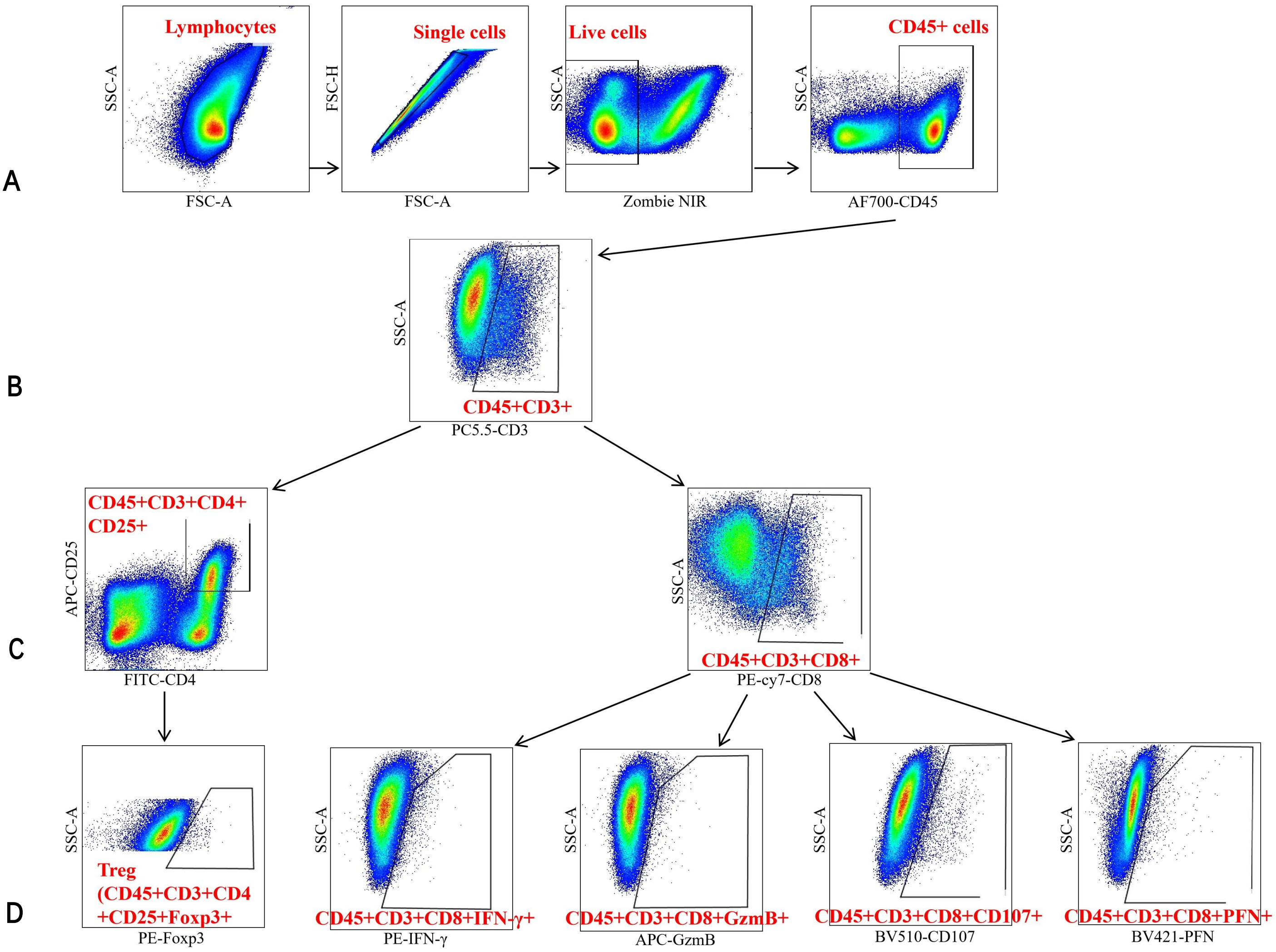

Supplementary Figure 3. Tumor-Infiltrating Leukocyte (TIL) Gating Strategy

(A) Single cells: From the forward scatter area (FSC-A) vs. height (FSC-H) plot, doublets and cell aggregates were excluded to select single cells. Live cells: Within the single-cell gate, live cells were identified by excluding dead cells (stained with Zombie NIRviability dye). CD45 cells: Live single cells were further analyzed to gate on total immune cells (CD45) using anti-CD45 (AF700) staining. (B) T cells: Within the CD45 gate, T cells were identified as CD3 (PC5.5-CD3). (C) CD4 T cells: CD3CD4 T cells were identified via FITC-conjugated anti-CD4 staining. CD3CD8 cells were identified via PE-Cy7-conjugated anti-CD8 staining. (D) regulatory T cells (Treg): among CD4+T cells Treg cells were characterized as CD25Foxp3 (APC-CD25 followed by PE-Foxp3). And further characterization of CD8+ T cells by IFN-γ: (PE-IFN-γ), Granzyme B (APC-GzmB), CD107 (BV510-CD107), and Perforin (BV421-PF4).
